# “Scientific Evidence for Homeopathy”

**DOI:** 10.1016/j.clinsp.2023.100255

**Published:** 2023-07-25

**Authors:** Marcus Zulian Teixeira

**Affiliations:** Departamento de Psiquiatria, Faculdade de Medicina, Universidade de São Paulo, São Paulo, SP, Brazil

## Abstract

• Homeopathy is based on heterodox and complementary four scientific assumptions.
• Pseudosceptics deny these assumptions and any scientific evidence that proves them.
• The dossier describes hundreds of clinical and experimental studies in homeopathy.
• The dossier demystifies the fallacy that “there isn't scientific evidence for homeopathy”.

Upon discussing homeopathy in various settings, the authors often find that people react with mistrust, and raise doubts about its scientific grounds and therapeutic validity. Widely disseminated in the mass media, in indistinct and reiterated manner, the fallacy – or post-truth – asserting “there isn't scientific evidence for homeopathy” is incorporated into the collective subconscious, thus serving as a strategy to increase prejudice and radicalize postures against this bicentennial medical approach.

A fruit of disinformation or of negation of the studies that ground the homeopathic paradigm on many scientific fields, prejudice is once and again fed by unfavorable pieces published in the mass media and social networks, which, in turn, very seldom divulge studies with results favorable to homeopathy.

Homeopathy has been a medical practice recognized worldwide for over two hundred years, developing care, teaching, and research activities in various health institutions and medical schools. It employs a clinical approach based on heterodox and complementary scientific assumptions (principle of cure by similarity, homeopathic pathogenetic trials, use of dynamized doses and individualized medicines), with the aim of triggering a curative response from the organism against its own disorders or illnesses.^1^

In view of being based on principles different from those employed by conventional medical practice, homeopathy is often the target of unfounded and widespread criticism by individuals who, systematically, deny homeopathic assumptions and any scientific evidence that proves them, as they are involved in dogmatic posture that prevents a correct and free analysis of prejudices. In reality, they are pseudo sceptics masquerading as pseudoscientists.

To elucidate physicians, researchers, health professionals, and the general public, demystifying culturally ingrained dogmatic postures and the pseudosceptical fallacy that “there isn't scientific evidence for homeopathy”, in 2017, the Technical Chamber for Homeopathy of the Regional Medical Council of the State of São Paulo (CREMESP, Brazil) prepared the Special Dossier “Scientific Evidence for Homeopathy”.^2^, ^3^

This project had the support of the Brazilian Homeopathic Medical Association (AMHB) and the São Paulo Homeopathic Medical Association (APH) via divulgation in its scientific journal, *Revista de Homeopatia* (São Paulo), in three independent editions: online in Portuguese,^4^ online in English^5^ and printed in Portuguese.^6^ Expanding its dissemination to the Spanish-speaking public, this dossier has just been published in the scientific journal *La Homeopatía de México* in a special edition of the journal's 90^th^ anniversary.^7^

In addition to describing the global situation of homeopathy as a medical specialty and its inclusion in the curricula of medical schools, the dossier further includes reviews on research lines that provide grounds to the homeopathic assumptions, to wit: therapeutic similitude principle, homeopathic pathogenetic trials, dynamized doses (High Dilutions – HDs), and medicine individualization based on the set of characteristic symptoms exhibited by patient/disease. Similarly, the efficacy and safety of homeopathic treatment are demonstrated in randomized, placebo-controlled clinical trials, systematic reviews, and meta-analyses.

The dossier begins with a review entitled “Homeopathy: a brief description of this medical specialty”,^8^, ^9^, ^10^ which discusses historical, social, and political aspects of the institutionalization of homeopathy in Brazil and its inclusion in health care systems. It further describes the reasons for patients to seek this therapeutic approach.

The review on “Medical education in non-conventional therapeutics in the world (homeopathy and acupuncture)”^11^, ^12^, ^13^ highlights the relevance of the inclusion of homeopathy and acupuncture in the curriculum of medical schools in many countries around the world. Such inclusion – actualized in various modalities specifically targeting undergraduate and graduate students, medical residents, and practicing doctors ‒ is a result of the increasing interest of patients, leading to a similar interest among doctors to learn about such medical approaches.

Looking to provide scientific grounds for the therapeutic similitude principle through a systematic study of the rebound effect of modern drugs, the review entitled “Scientific basis of the homeopathic healing principle in modern pharmacology”^14^, ^15^, ^16^ discusses hundreds of studies published in high-impact scientific journals, which demonstrate a conceptual and phenomenological similarity between rebound effect and the vital reaction (or secondary action) homeopathic treatment elicits. Aiming at broadening the implications of such similarity, the author describes the use of modern drugs according to the therapeutic similitude principle, which leads to the application of the rebound effect (paradoxical reaction of the organism) with curative intention.

To account for the plausibility of the homeopathic use of HDs, the present dossier includes three reviews that describe the advances made in fundamental research over the past decades: “The soundness of homeopathic fundamental research”,^17^, ^18^, ^19^ “Effects of homeopathic high dilutions on *in vitro* models: a literature review”,^20^, ^21^, ^22^ and “Effects of homeopathic high dilutions on plants: a literature review”.^23^, ^24^, ^25^ These reviews discuss hundreds of experiments and dozens of lines of research that together demonstrate the effects of HDs in physical-chemical and biological models (*in vitro*, plants and animals).

Demonstrating that the positive effects of homeopathic treatment "are not a mere placebo effect", as it is widely advertised, the review “Clinical research in homeopathy: systematic reviews and randomized clinical trials”^26^, ^27^, ^28^ describes the positive results found in dozens of homeopathic placebo-controlled clinical trials targeting variable clinical conditions, as well as systematic reviews and meta-analyses. These results are particularly illustrated by two clinical trials conducted at prestigious Brazilian research institutions: “Potentized estrogen in the homeopathic treatment of endometriosis-associated pelvic pain: A 24-week, randomized, double-blind, placebo-controlled study”^29^, ^30^, ^31^ and “Randomized, double-blind trial on the efficacy of homeopathic treatment in children with recurrent tonsillitis”.^32^, ^33^, ^34^

As concerns the safety of homeopathic treatment, the review entitled “Do homeopathic medicines cause drug-dependent adverse effects or aggravations?”^35^, ^36^, ^37^ demonstrates, through an analysis of placebo-controlled clinical trials, that although mild and transient, homeopathic medicines cause more adverse effects compared to placebo.

The final review, “Do homeopathic medicines induce symptoms in apparently healthy volunteers? The Brazilian contribution to the debate on homeopathic pathogenetic trials”^38^, ^39^, ^40^ discusses the historical development and the state of the art in homeopathic pathogenetic trials. These experiments are conducted to establish the curative properties of drugs (pathogenetic effects on healthy individuals) that ground the application of the therapeutic similitude principle.

Despite the ongoing difficulties and limitations opposing the development of research in homeopathy – partly due to methodological aspects, and partly to lack of institutional and financial support – the experimental and clinical studies described in this dossier, which ground the homeopathic assumptions and confirm the efficacy and safety of this approach to therapeutics – provide unquestionable proof for the “availability of scientific evidence for homeopathy”, against the prejudice falsely disseminated by pseudosceptics and pseudoscientists.^41^^,^^42^

With the divulgation of the present dossier at open access trilingual editions, the authors hope to dispel doubts and sensitize their colleagues as to the validity and relevance of homeopathy as an adjuvant treatment complementary to all other medical specialties according to ethical and safe principles. In conformity with this integrative approach, homeopathic practice allows to broaden the understanding of human disease, increase therapeutic resources, contribute to the definition and effectiveness of medicine in chronic diseases, minimize the adverse effects of modern drugs and strengthen the patient-doctor relationship, among other aspects.

## Funding

This research did not receive any specific grant from funding agencies in the public, commercial, or not-for-profit sectors.

## Declaration of Competing Interest

The authors declare no conflicts of interest.
